# The ASPECT hydrocephalus system: investigating clinical applicability and system utility

**DOI:** 10.1007/s00701-024-06274-w

**Published:** 2024-10-09

**Authors:** N M Toft, S Hornshøj Pedersen, T S R Jensen, J Birch Milan, C S Riedel, N Agerlin, P Birkeland, J Hauerberg, C C Larsen, T N Munch, A Vedel Holst, M Juhler

**Affiliations:** 1 CSF Study Group, Copenhagen, Denmark; 2 Department of Neurosurgery, Copenhagen University Hospital (Rigshospitalet), Copenhagen, Denmark; 3 Department of Radiology, Copenhagen University Hospital (Rigshospitalet), Copenhagen, Denmark; 4 Department of Clinical Medicine, University of Copenhagen, Copenhagen, Denmark

**Keywords:** Hydrocephalus, ASPECT Hydrocephalus System

## Abstract

**Purpose:**

Hydrocephalus presents diagnostic and management challenges due to its heterogeneity. The ASPECT Hydrocephalus System, introduced in 2023, offers a comprehensive approach to describing patients with hydrocephalus. This study investigates the clinical applicability of the ASPECT Hydrocephalus System compared to the International Classification of Disease (ICD-10) and demonstrates its utility.

**Methods:**

Two hundred pediatric and adult patients with hydrocephalus treated at Copenhagen University Hospital between September 2019 and 2020 were described according to the ASPECT Hydrocephalus System. The latest brain imaging served as assessment point.

**Results:**

Forty-seven percent of patients had more than one ICD-10 code assigned, and 40.5% of patients had an unspecific ICD-10 code as the most recent. It was possible to apply factor A (anatomy), S (symptomatology), P (previous interventions), C (complications) and T (time of onset) to all patients. Factor E (etiology) categorized 15% of patients as ‘unknown’. Combining factor A and S showed a similar incidence of acute high-pressure symptoms in patients with and without ventriculomegaly on imaging (39.7% vs 39.3%), demonstrating how symptoms and neuro-radiological findings do not necessarily correlate.

**Conclusion:**

The ASPECT Hydrocephalus System’s applicability and utility were demonstrated in a large, diverse patient population. Except for ‘Etiology’, all factors could be applied to the entire population showing the system’s robustness. While limitations in ICD-10 may force clinicians to choose between clinical measures, the ASPECT Hydrocephalus System allows comprehensive patient characterization, potentially aiding in clinical decision-making and research. Its use depends on registration quality. Application in prospective cohorts is warranted to assure feasibility.

**Supplementary Information:**

The online version contains supplementary material available at 10.1007/s00701-024-06274-w.

## Introduction

Hydrocephalus is a complex and multifactorial disorder with variations in patient characteristics, patoanatomy, clinical presentation, etiology, and prognosis, making treatment and management of each patient a multifaceted task [13]. A precise diagnostic description is therefore an important part of disease management. The International Classification of Diseases (ICD) [6] is used worldwide to classify patients with hydrocephalus and categorizes patients based on anatomy, underlying etiology, or clinical symptomatology (e.g., normal pressure hydrocephalus [NPH]).

Previously published alternatives to the ICD classification system characterize hydrocephalus by either one or two patient-related factors: communication vs non-communication between ventricular compartments [5], specifying point of obstruction [11], etiology and time of onset [9, 12], or pathophysiology [2, 10]. However, none of these systems are widely accepted or generally used [11].

In 2023, a multifactor non-hierarchic descriptive system, the ASPECT Hydrocephalus System, was published [8]. The system was developed in collaboration between the Copenhagen CSF Study Group and the CSF task force of the European Association of Neurosurgical Societies and aims to provide a simple and applicable tool encompassing the complexity of patients with hydrocephalus in both a clinical and scientific setting. The ASPECT Hydrocephalus System consists of six different patient-related factors important to prognosis, clinical decision-making, and research (Table 1) [8].

**Table 1:** ASPECT Hydrocephalus system. Optional answer for all factors:”9″ if answer is unknown. ‘Acute high-pressure symptoms’ include acute severe headache, vomiting, nausea, reduced consciousness, visual loss, oculomotor disturbances, other focal deficits and seizures. For infants acute high-pressure symptoms are: enlarged head size, frontal bossing, prominent scalp veins, sun-setting eyes, nystagmus and wide and tense anterior fontanelle. ‘Other acute symptoms’ are less severe pressure symptoms than acute high-pressure symptoms, e.g. frequent symptoms such as increasing light to moderate headache, nausea and fatigue and less frequent symptoms such as affected consciousness, impaired vision and motor symptoms such as eye movement symptoms. ‘Chronic symptoms’ are mostly described in the context of NPH as Hakims triad (gait instability, urinary incontinence and dementia). However, a constellation of cognitive and motor-symptoms are also typical of chronic hydrocephalus in younger age groups. Chronic symptoms in the ASPECT Hydrocephalus System are categorized as stable or progressive based on the patient’s medical history. Patients with improvement in their symptomatology since last medical evaluation are categorized as ‘improved symptoms’. Distribution of patients in all six ASPECT factors: Anatomy has 600 entries as every patient is assessed in three categories. The factors Symptomatology, Etiological factors, and Time of onset only has one entry per patient. The patient may have several entries in Previous interventions and Complications to previous interventions. ETV: endoscopic third ventriculostomy. EVD: external ventricular drain, ICP: intra cranial pressure. *Atypical ventriculomegaly might be observed in cases of, e.g., multiloculated hydrocephalus [1]. †Obstruction, disconnection, tube defect and valve dysfunction

ASPECT factor	Total, n (%)
Anatomy, n (%)	200/200 (100)
* Ventricles*	
Normal	38/200 (19)
Reduced/overdrained	22/200 (11)
With slit ventricles	11/22 (50)
With subdural hematoma/hygroma	6/22 (27.3)
Ventriculomegaly	141/200 (70.5)
Unilateral	12/141 (8.5)
Both lateral ventricles	20/141 (14.2)
Both lateral ventricles and 3rd	75/141 (53.2)
All ventricles	32/141 (22.7)
Difference between lateral ventricles	4/200 (2)
* Subarachnoid space*	
Unaffected subarachnoid space	135/200 (67.5)
Compressed subarachnoid space	41/200 (20.5)
Enlarged subarachnoid space	24/200 (12)
DESH	12/200 (6)
* Additional*	
None	131/200 (65.5)
Cystic	21/200 (10.5)
Atypical *	48/200 (24)
Symptomatology, n (%)	200/200 (100)
Asymptomatic	57/200 (28.5)
Acute high pressure	37/200 (18.5)
Other acute	43/200 (21.5)
Chronic progressive	34/200 (17)
Chronic stable	19/200 (9.5)
Improved	10/200 (5)
Previous intervention (multiple options possible), n (%)	200/200 (100)
None	18/200 (9)
Shunt	147/200 (73.5)
ETV	53/200 (26.5)
EVD	64/200 (32)
ICP monitoring	37/200 (18.5)
Other	0/200 (0)
Etiological factors, n (%)	200/200 (100)
Developmental or genetic anomaly	77/200 (38.5)
Infection	5/200 (2.5)
Vascular	47/200 (23.5)
Neoplasm	29/200 (14.5)
Trauma	12/200 (6)
Unknown	30/200 (15)
Complications to previous interventions(multiple options possible), n (%)	200/200 (100)
None	105/200 (52.5)
Mechanical shunt failure †	87/200 (43.5)
ICP malregulation with functional shunt	32/200 (16)
Functional ETV failure	3/200 (1.5)
* Surgical complication unrelated to shunt function*	
Infection	15/200 (7.5)
Bleeding	2/200 (1)
Skin defect	0/200 (0)
Shunt displacement	0/200 (0)
CSF leakage	0/200 (0)
* Patient related complications unrelated to shunt function*	
Shunt related pain	0/200 (0)
Allergies	1/200 (0.5)
Other	0/200 (0)
Time of onset, n (%)	200/200 (100)
Fetal/congenital	65/200 (32.5)
Pediatric hydrocephalus	17/200 (8.5)
Suspected congenital/pediatric in adult	19/200 (9.5)
Adult hydrocephalus	99/200 (49.5)

The aim of this study was to investigate 1) clinical applicability in comparison with the ICD-10 classification system, and 2) utility of the ASPECT Hydrocephalus System.

## Methods

### Study population

The ASPECT Hydrocephalus System was applied to a retrospective cohort of consecutive non-selected patients comprising all patients with hydrocephalus treated at the Department of Neurosurgery at Copenhagen University Hospital from September 2019 to September 2020. We excluded patients with later disconfirmed hydrocephalus and patients without an available brain imaging within the inclusion period.

### The ASPECT hydrocephalus system

The ASPECT Hydrocephalus system consists of six clinical factors: A (anatomy) is cerebral anatomy based on computed tomography (CT) or magnetic resonance imaging (MRi); S (symptomatology) is symptoms sub grouped into acute and chronic presentations; P (previous interventions) is a comprehensive list of all surgical previous interventions; E (etiology) categorizes patients by cause of hydrocephalus; C (complications) is a list of complications to all previous interventions, and T (time of onset) is time of disease onset. Factors appear in a non-hierarchical order. Each factor has a set of predefined, numbered answers that allow for the application of a standardized numerical code to patients (Table 1).

### Data extraction

Electronic health records were retrospectively reviewed. The latest recorded ICD-10 code and the total number of ICD-10 codes related to hydrocephalus from the time of initial diagnosis to the time of latest brain imaging, were registered for each patient. The diagnoses G91.9 hydrocephalus, unspecified; G91.8 hydrocephalus, other; Q03.9 congenital hydrocephalus, unspecified; and G94.2 hydrocephalus classified in disease elsewhere were subcategorized as unspecific diagnoses.

The time of the latest CT or MRi was chosen as the time point for our current assessment of the ASPECT Hydrocephalus System; i.e., all other factors were related to this time point. Factor A was rated by a neurosurgeon subspecialising in hydrocephalus. Clinical information and neurological examination, conducted by either a neurosurgeon, a neurologist, or a neuro-pediatrician within 45 days plus/minus of the CT or MRi was used to assess factor S. Further information on previous neurosurgical interventions, etiology, complications related to previous interventions, current age and time of onset were extracted to assess factor P–T.

If a patient did not match one of the predefined categories in the ASPECT Hydrocephalus System, e.g., if essential information was missing, or test results were divergent, the given factor was categorized as ‘Unknown’.

### Statistics

Data were stored in a REDCap database and data analysis was conducted using RStudio (V.3.1.3). Data are presented as number of cases and percentages.

## Results

### Study population

**Fig. 1:**
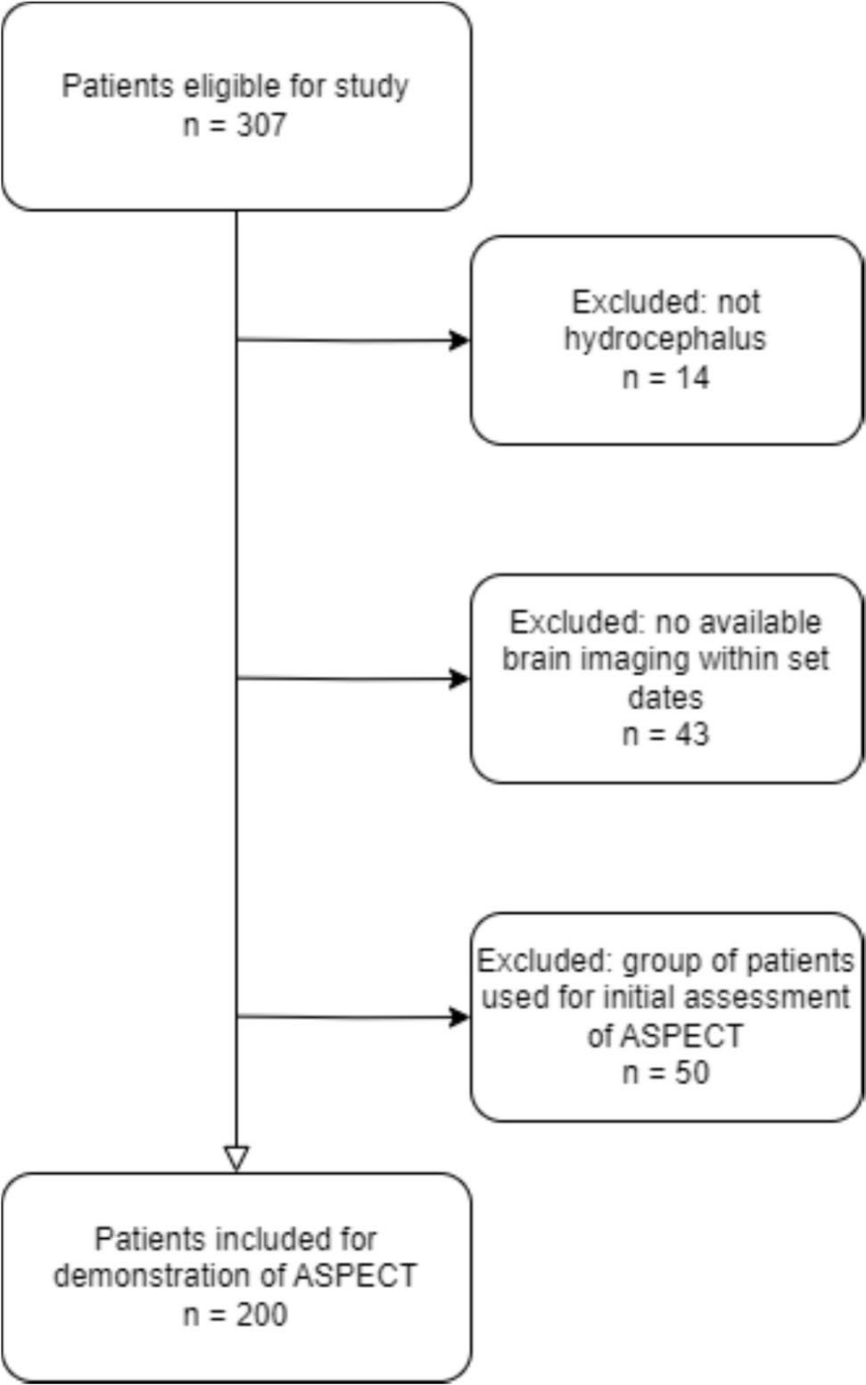
Flowchart of the patient inclusion process

Of 307 eligible patients, 200 patients were included in the study (male 50%, adults 73%) (Fig. 1). The paediatric patients were distributed as 19 males (mean age: 8.1 years; range: 0–17 years) and 24 females (mean age: 6.4 years; range 0–17 years). Distribution of the adult patients were 81 males (mean age: 53.7 years; range: 18–84 years) and 76 females (mean age: 54 years; range: 18–85 years).

### Clinical applicability

#### The international classification of diseases

**Fig. 2:**
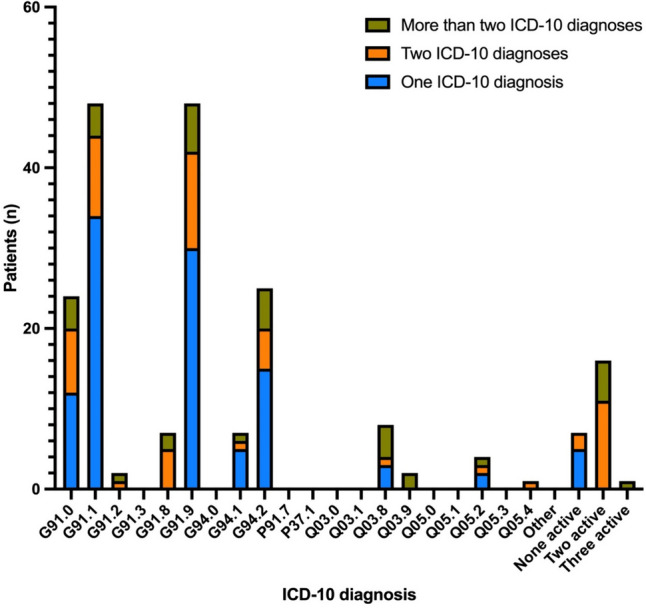
Active ICD-10 codes for patients with one, two or more than two ICD-10 codes. The four most frequent ICD-10 codes of the 106 patients that was assigned only one ICD-10 code was: G91.1 obstructive hydrocephalus (n = 34; 53,2%), G91.9 hydrocephalus, unspecified (n = 30; 54,53%), G94.2 hydrocephalus classified in disease elsewhere (n = 15; 23,94%) and G91.0 communicating hydrocephalus (n = 12; 15,96%). Of the patients assigned more than one ICD-10 code, 29% were assigned two ICD-10 codes, 12% three ICD-10 codes, and 6% four or more ICD-10 codes. Twenty-five of the patients (6%) with more than one ICD-10 code were given G91.9 (hydrocephalus, unspecified) or G91.8 (hydrocephalus other) as the primary ICD code

Of the 200 included patients, nearly half were assigned more than one ICD-10 code (Fig. 2). For patients with only one ICD-10 code (106/200), the most frequent unspecific diagnosis was G91.9 (16/106), while the most frequent specific diagnosis was G91.1 (18/106).

For patients with two or more ICD-10 codes (94/200), 61/94 patients had an unspecific diagnosis combined with a specific diagnosis, while 22/94 patients had several specific diagnoses, e.g., 8/94 patients were diagnosed with both G91.0 (communicating hydrocephalus) and G91.1 (obstructive hydrocephalus).

In 81 of the 200 included patients the latest ICD-10 code was an unspecific diagnosis: G91.8; G91.9; Q03.9 or G94.2. Forty-five of these had one unspecific diagnosis, while 9/81 had multiple unspecific diagnoses. The remaining 27/81 patients had a specific diagnosis prior to the unspecific diagnosis.

#### The ASPECT hydrocephalus system

The distribution of the ASPECT-factors in the cohort are shown in Table 1. It was possible to apply factor A, S, P, C and T to all included patients. In factor E (etiology) 15% of the patients were categorized as ‘unknown’ all of which were adults.

#### Utility of the ASPECT hydrocephalus system

The ASPECT Hydrocephalus System is not intended as a classification system. However, the descriptive factors may enable categorization of a population based on either a single factor or a combination of several factors. Examples of this utility are shown below.

#### Combining factor A (anatomy) and S (symptomatology)

The relevance of combining factors A and S could be to e.g., correlate neuro-radiological findings to the symptomatology.

The majority of patients had ventriculomegaly on their brain imaging (141/200), with enlargement of both lateral ventricles and 3rd ventricle as the most common subgroup (75/141). However, only 56/141 patients with ventriculomegaly had a presentation of acute high-pressure symptoms or other acute symptoms, while 23/141 had progressive chronic symptoms and 17/141 had stable chronic symptoms. One-fourth (37/141) were asymptomatic and 8/141 had improved symptoms since last examination.

Twenty-eight out of 141 patients had brain imaging with normal ventricles, unaffected subarachnoid space and no additional abnormalities. However, only 9/28 of these patients were asymptomatic, while 11/28 had acute high-pressure symptoms or other acute symptoms.

Hence, combining factor A and S illustrates how symptoms do not always align with neuro-radiological findings.

#### Combining factor P (previous interventions), E (etiology) and C (complications)

It may be relevant to combine factors P, E and C to examine e.g., the risk of complications related to etiology or previous shunt history. The main previous interventions were shunt treatment and ETV. Shunt treatment was performed in nearly three-quarters (147/200) of patients while ETV was performed in 53/200 patients. ETV was the only surgical intervention in 28/200 instances. At the time of study inclusion, 18/200 of the patients had not received any neurosurgical treatment.

Of the 147 patients treated with a shunt, 106/147 had multiple shunt surgeries. The correlation between shunt surgeries, surgically related complications, and etiology is listed in Table 2. Patients with a history of more than 5 previous shunt surgeries (27/147) had a higher frequency of surgical complications, e.g., mechanical shunt failure (100% vs. 65.4–85.2%) and infection (35.7–38.5% vs. 3.7–7.7%) compared to patients with 2–3 and 4–5 shunt surgeries, respectively (Table 2). The main etiology in this patient group (more than 5 shunt surgeries) was ‘Developmental or genetic anomaly’ compromising 69.2–85.7% of the patients. The 27 patients who had more than 5 previous shunt surgeries were aged 3–66 years (mean age: 27.7 years) with time of onset being primarily fetal/congenital (20/27; 74.1%). In this subgroup the total number of surgeries ranged from 6–21, corresponding to 9.2 surgeries pr. patient.

**Table 2:** Patients with shunt surgeries in five groups, with distribution of etiologies and complications. Time and date were not logged for previous interventions or complications, although these could be of interest when comparing patients

Shunt surgeries, etiology and complications					
Shunt surgeries (n)	1	2–3	4–5	6–7	≥ 8
Patients, n (%)	41/147 (27.9)	52/147 (35.4)	27/147 (18.4)	13/147 (8.8)	14/147 (9.5)
Etiology					
Developmental or genetic anomaly, n (%)	10/41 (24.4)	16/52 (30.8)	9/27 (33.3)	9/13 (69.2)	12/14 (85.7)
Infection, n (%)	1/41 (2.4)	2/52 (3.8)	0/27 (0)	0/13 (7.7)	0/14 (0)
Vascular, n (%)	14/41 (34.1)	13/52 (25)	11/27 (40.7)	1/13 (7.7)	1/14 (7.1)
Neoplasm, n (%)	9/41 (22)	11/52 (21.2)	2/27 (7.4)	1/13 (7.7)	1/14 (7.1)
Trauma, n (%)	2/41 (4.9)	3/52 (5.8)	3/27 (11.1)	1/13 (7.7)	0/14 (0)
Unknown, n (%)	5/41 (12.2)	7/52 (13.5)	2/27 (7.4)	1/13 (7.7)	0/14 (0)
Complications					
None, n (%)	37/41 (90.2)	12/52 (23.1)	3/27 (11.1)	0/13 (0)	0/14 (0)
Mechanical shunt failure, n (%)	3/41 (7.3)	34/52 (65.4)	23/27 (85.2)	13/13 (100)	14/14 (100)
ICP malregulation with functional shunt, n (%)	0/41 (0)	10/52 (19.2)	9/27 (33.3)	4/13 (30.8)	9/14 (64.3)
Functional ETV failure, n (%)	0/41 (0)	2/52 (3.8)	1/27 (3.7)	0/13 (0)	0/14 (0)
*Surgical complication unrelated to shunt function*					
Infection, n (%)	0/41 (0)	4/52 (7.7)	1/27 (3.7)	5/13 (38.5)	5/14 (35.7)
Bleeding, n (%)	0/41 (0)	1/52 (1.9)	0/27 (0)	0/13 (0)	1/14 (7.1)
Skin defect, n (%)	0/41 (0)	0/52 (0)	0/27 (0)	0/13 (0)	0/14 (0)
Shunt displacement, n (%)	0/41 (0)	0/52 (0)	0/27 (0)	0/13 (0)	0/14 (0)
CSF leakage, n (%)	0/41 (0)	0/52 (0)	0/27 (0)	0/13 (0)	0/14 (0)
*Patient related complications unrelated to shunt function*					
Shunt related pain, n (%)	0/41 (0)	0/52 (0)	0/27 (0)	0/13 (0)	0/14 (0)
Allergies, n (%)	0/41 (0)	0/52 (0)	0/27 (0)	0/13 (0)	1/14 (7.1)

One third of the patients who had shunt surgery (52/147) did not have a prior history with complications related to shunt treatment. The etiologies in this patient group were distributed as vascular (16/52), developmental or genetic anomaly (12/52), neoplasm (11/52), unknown (6/52), trauma (5/52), and neuroinfection (2/52).

Patients with ICP malregulation (32/200) all had a history with at least 2 shunt surgeries (range: 2–20 surgeries) and 13 of these had more than 5 previous shunt surgeries. Half of these patients (17/32) were diagnosed with a developmental or genetic anomaly. Only 1 patient experienced underdrainage, while the remaining had suffered from overdrainage.

#### Combining factor P (previous interventions), C (complications) and T (time of onset)

The relevance of combining factors P, C and T could be to relate complication and revision risks to time of onset and hydrocephalus duration.

Patients with fetal or congenital hydrocephalus had more shunt surgeries and were more likely to suffer a mechanical shunt failure (4.6 shunt surgeries and 1.9 shunt failures per patient), compared to patients with paediatric onset (2.5 shunt surgeries and 1.1 shunt failures per patient), suspected congenital or pediatric onsetdiagnosed as an adult (0.4 shunt surgeries and 0.2 shunt failures per patient) or adult onset (1.9 shunt surgeries and 0.6 shunt failure per patient).

#### Combining factor E (etiology) and ICD-10 codes

All options in the factor E were present both in the group with ICD-10 code G91.0 (27/200) and in the group with ICD-10 code G91.1 (56/200). The distribution of etiologies was comparable in these two ICD-10 subgroups for the following etiological categories: developmental or genetic anomaly (40.7% vs. 41.1%), vascular causes (18.5% vs. 19.6%), and infection (3.7% vs. 1.8%).

More patients in the G91.1 subgroup had an unknown etiology compared to the G91.0 subgroup (19.4% vs. 3.7%). Interestingly, both hydrocephalus due to trauma and due to neoplasm were more likely in the G91.0 subgroup (11.6% vs. 3.6%, and 22.2% vs. 14.3%).

## Discussion

With this study, we 1) elaborate clinical applicability of the ASPECT Hydrocephalus System in comparison with the ICD-10 classification system, and 2) demonstrate utility of the ASPECT Hydrocephalus System. Several of the 200 included patients had multiple ICD-10 codes, unspecific ICD-10 codes, or conflicting ICD-10 codes. Except for the factor ‘Etiology’, which was applicable to 85%, the five other factors in the ASPECT Hydrocephalus System could be applied to the entire study population. It was anticipated that some patients would have an unknown etiology, as the cause and pathophysiology remain unclear in certain cases and variants of hydrocephalus.

### Clinical applicability

#### The international classification of diseases

Though the ICD classification system has grown to capture more of the variability in hydrocephalus, it still has important limitations. ICD-10 diagnoses related to hydrocephalus are based on anatomy (obstructive vs. communicating), underlying etiology, or clinical symptomatology (e.g., NPH), and, as such, this system forces the clinician to choose between these, although information on anatomy, etiology, and symptoms would apply to any patient. As a consequence of this, one patient can receive more than one specific ICD-10 diagnosis, which was the case in 21% of our population. Also, patients with different types of hydrocephalus may receive identical ICD-10 diagnoses. A classification system in which one patient may have multiple appropriate diagnoses while other patients only receive a non-specific diagnosis is of limited clinical value [8]. The precise cause for a notable prevalence of unspecific ICD-10 diagnoses in this study is unknown, and we can only hypothesize about the potential causes. Cases with one diagnosis could be patients with hydrocephalus with unknown etiology, patients in the process of examinations to determine cause or patients with no suitable ICD-10 code. The reason for altered diagnoses in 27 cases, may be due to a primary misdiagnosis, though this is unlikely the cause in every case. A contributing factor might be redundancy of choice, which serves little purpose for the patient or the clinician. Regardless of the underlying causes, 81 patients had unspecific diagnoses at the time data was collected, which means 40.5% of the included population had an ICD-10 diagnosis, which provided no meaningful information about their disease.

#### The ASPECT hydrocephalus system

Within the factor ‘Etiology’, 15% of patients were categorized as ‘Unknown’. In comparison, one study [12] with a population of 517 adults with hydrocephalus found that 38.7% of patients had idiopathic NPH (iNPH) and 5% had hydrocephalus with an unknown etiology. The mean age in the study was 58.9 years (± 19.3), which could explain the high number of iNPH [4, 7]. The ASPECT Hydrocephalus System covers patients with all hydrocephalus types across all ages, as it was possible in all instances to categorize patients in a specific category in each of the remaining five factors. Direct causes of hydrocephalus may be bleeding, trauma, infection, tumor, or anatomical malformations detectable with cerebral imaging, however complete etiological classification of all patients with hydrocephalus is utopian, particularly when there is a considerable timespan between the onset of the underlying etiology and the hydrocephalus diagnosis and/or initiation of treatment. The pathology of idiopathic cases of hydrocephalus is not well understood and is still being investigated [3]. In complex cases with competing causes, e.g., a trauma patient with an infected EVD, it is hard to tell which is the primary cause or if both are contributing factors to the development of hydrocephalus. Therefore, these challenges may not be aided by the ASPECT Hydrocephalus System.

### System utility of the ASPECT hydrocephalus system

Several ASPECT factors from the same patient form a timeline of the patient’s history and could be useful in clinical assessment and treatment planning. The data collected from these timelines could potentially be valuable in research as well. The traits of the ASPECT Hydrocephalus System, with the ability to group patients by selected factors, could make it a valuable tool for focusing on specific hydrocephalus characteristics. Such research into distinctive groups of patients with hydrocephalus could help acquire knowledge to further specialize and personalize management and treatment. *Williams *et al*.* [12] described demographics and clinical characteristics of 517 adults with hydrocephalus including etiology, symptoms, and treatment. Our cohort is somewhat comparable to the one in the work of *Williams *et al*.* as several of the same factors are used in categorizing patients. Patients of the *Williams *et al*.* cohort were categorized in four groups: ‘acquired’ were adults with e.g. vascular, trauma, neoplasm; ‘transition’ was adult patients with pediatric/suspected pediatric onset hydrocephalus; ‘unrecognized hydrocephalus’ was mostly asymptomatic adults with incidentally discovered hydrocephalus caused by developmental anomalies and ‘suspected iNPH’ was untreated patients with signs and symptoms consistent with iNPH. The paper compared symptoms and impairments across the groups, finding ‘unrecognized hydrocephalus’ to be the one group with least impairments or etiologies leading to neurological injury. These groups were based on different clinical characteristics, e.g., time of onset, a specific set of symptoms or unrecognized disease. ‘Suspected iNPH’ was the most homogenous group on age and symptoms, remaining groups varies more on age, symptoms, and etiology. The authors found an overlap of patients in ‘transition’ and ‘unrecognized hydrocephalus’ who share the same etiologies but differ in time of onset and severity of symptoms. These differences in hydrocephalus-related factors within each distinct group are not necessarily a problem, but they could be in certain situations. To study patients with a disease as many-faceted as hydrocephalus, homogenous groups of patients would in some instances be preferred. Mixing subgroups in a study would make analysis and interpretation harder and might not reveal important trends and connections. Findings derived from such a study could not be applied universally within the group or patient population of interest. Homogenous groups have less variables making them easier to study independently and compare to other groups. This approach would make it easier to reveal treatments and interventions effect on outcome in certain subgroups. With the ASPECT Hydrocephalus System it is possible to extract groups of homogenous patients, as patients can be grouped according to one or more specific factors of interest.

### Strength and limitations

The system depends on the quality of the obtained patient history, physical exam, and results from clinical investigations. Thorough and correct registration is necessary. Initial use of the ASPECT Hydrocephalus System in a department would initially require time from surgeons to obtain historic data for factors P and C on each patient upon their first visit. Subsequently, in our experience, new entries by next visit should only take minutes. We expect that medical doctors working with hydrocephalus patients will be able to use the ‘Anatomy’ factor with training. The large and diverse population of 200 patients with hydrocephalus contained both pediatric and adult patients, acute and chronic patients, and a representative distribution of etiology resulted in a thorough examination of all factors in the ASPECT Hydrocephalus System. Although we expect that the frame with the defined factors is robust, we also expect that a prospective study on larger cohorts in different centers may reveal minor deficiencies that need to be corrected. To further evaluate the ASPECT Hydrocephalus System, an inter-rater and intra-rater reliability study should be conducted to measure consistency in its use among different clinicians as well as repeated use by the same clinician. In this study it was not possible to evaluate either of these parameters.

## Conclusion

With this study we demonstrate the applicability and utility of the ASPECT Hydrocephalus System in a diverse patient population, showcasing its robustness in characterizing patients with hydrocephalus across various factors. While the International Classification of Diseases (ICD-10) system offers some categorization, it falls short of capturing the complexity of hydrocephalus, often leading to unspecific diagnoses and limited clinical value. The ASPECT Hydrocpehalus System, however, provides a comprehensive framework for patient characterization, potentially aiding clinical decision-making and research.

## Supplementary Information

Below is the link to the electronic supplementary material.

Supplementary file1 (DOCX 18 KB)

## Data Availability

Data is provided within the manuscript or supplementary information files
