# Supplementary material for: The ASPECT hydrocephalus system: investigating clinical applicability and system utility
Source: Acta Neurochir (Wien). 2024 Oct 9;166(1):401. doi: 10.1007/s00701-024-06274-w (PMC11464604; doi:10.1007/s00701-024-06274-w)
Supplement: Supplementary file 1 — Supplementary file1 (DOCX 18 KB) [file 701_2024_6274_MOESM1_ESM.docx]

## **Supplementary**

| **Previous interventions** | **Total, n (%)** |
| --- | --- |
| **None,** n (%)  **Shunt,** n (%)  1 surgery  2-3 surgeries  4-5 surgeries  6-7 surgeries  ≥8 surgeries  **ETV,** n (%)  1 surgery  2 surgeries  3 surgeries  **EVD,** n (%)  1 surgery  2 surgeries  3 surgeries  **ICP monitoring,** n (%)  **Other,** n (%) | **18/200 (9)**  **147/200 (73.5)**  41/147 (27.9)  52/147 (35.4)  27/147 (18.4)  13/147 (8.8)  14/147 (9.5)  **53/200 (26.5)**  47/53 (88.7)  4/53 (7.5)  2/53 (3.8)  **64/200 (32)**  39/64 (60.9)  18/64 (28.1)  6/64 (9.4)  **37/200 (18.5)**  **0/200 (0)** |

Table 3. Number of interventions.

| **Complications to previous interventions,** number of complications | **Total,** n (%) |
| --- | --- |
| **None**  **Mechanical shunt failure,** n (%)  1  2-3  4-5  6-7  8-9  10-11  **ICP malregulation with functional shunt,** n (%)  **Functional ETV failure,** n (%)  1  **Surgical complication unrelated to shunt function,** n (%)  Infection  Bleeding  Skin defect  Shunt displacement  CSF leakage  **Patient related complications unrelated to shunt function,** n (%)  Shunt related pain  Allergies  Others | **105/200 (52.5)**  **87/200 (43.5)**  43/87 (49.4)  29/87 (33.3)  8/87 (9.2)  5/87 (5.7)  1/87 (1.1)  1/87 (1.1)  **32/200 (16)**  **3/200 (1.5)**  3/3 (100)  **16/200 (8)**  15/16 (93.8)  2/16 (12.5)  0/16 (0)  0/16 (0)  0/16 (0)  **1/200 (0.5)**  0/1 (0)  1/1 (100)  0/1 (0) |

Table 4. Number of complications.
